# Electric Field-Driven Assembly of Sulfonated Polystyrene Microspheres

**DOI:** 10.3390/ma10040329

**Published:** 2017-03-23

**Authors:** Alexander Mikkelsen, Jarosław Wojciechowski, Michal Rajňák, Juraj Kurimský, Khobaib Khobaib, Ahmet Kertmen, Zbigniew Rozynek

**Affiliations:** 1 Faculty of Physics, Adam Mickiewicz University, Umultowska 85, 61-614 Poznań, Poland; alexam@amu.edu.pl (A.M.); khobaib@amu.edu.pl (K.K.); 2 Department of Chemistry, University of Warsaw, Ludwika Pasteura 1, 02-093 Warsaw, Poland; jaroslaww@chem.uw.edu.pl; 3 Institute of Experimental Physics SAS, Watsonova 47, 040-01 Kosice, Slovakia; rajnak@saske.sk; 4 Faculty of Electrical Engineering and Informatics, Technical University of Košice, Letná 9, 04200 Košice, Slovakia; juraj.kurimsky@tuke.sk; 5 Faculty of Chemistry, Gdańsk University of Technology, Narutowicza 11/12, 80-233 Gdańsk, Poland; ahmet.kertmen@pg.gda.pl

**Keywords:** microparticles, sulfonation, spherical polystyrene particles, electric fields, self-assembly, electric conductance, dielectric constant, electro-rheology, droplets

## Abstract

A designed assembly of particles at liquid interfaces offers many advantages for development of materials, and can be performed by various means. Electric fields provide a flexible method for structuring particles on drops, utilizing electrohydrodynamic circulation flows, and dielectrophoretic and electrophoretic interactions. In addition to the properties of the applied electric field, the manipulation of particles often depends on the intrinsic properties of the particles to be assembled. Here, we present an easy approach for producing polystyrene microparticles with different electrical properties. These particles are used for investigations into electric field-guided particle assembly in the bulk and on surfaces of oil droplets. By sulfonating polystyrene particles, we produce a set of particles with a range of dielectric constants and electrical conductivities, related to the sulfonation reaction time. The paper presents diverse particle behavior driven by electric fields, including particle assembly at different droplet locations, particle chaining, and the formation of ribbon-like structures with anisotropic properties.

## 1. Introduction

Nano- and micrometer particles have become very important in today’s technological world. Though not visible to the naked eye, they interact and dictate mechanisms in various systems, ranging from cosmetic products to food. Due to their small size and large numbers, the self-organization and assembly of particles are required to further develop new technology which is reliant on smaller structures and complex materials.

Droplets are important in a vast range of areas, both in nature and industrially, involving phenomena such as capillarity, wetting, hydrodynamics, and materials such as emulsions and colloidosomes [1,2,3]. By decreasing the interfacial free energy of the system, particles find it energetically favorable to adsorb to fluid-fluid interfaces of droplets [4]. Particles may move in the plane of the interface, but movement normal to the interface is limited. Such a configuration is useful for numerous studies, including an experimental model system for basic questions of statistical mechanics [4,5], material development [6,7,8], particle structuring [9], and stabilizing emulsions by preventing drop coalescence [10,11,12]. The assembly of particles on drops is particularly attractive for the development of materials due to the architectural control it offers, together with the wide range of particles and solvents that can be used. Droplets covered with particles have been studied in different setups, including shear flows, where the particles affect drop deformation, inclination, and relaxation [13,14,15], and in magnetic or electric fields for manipulating fluids and particle dynamics [16,17,18,19,20].

In this work, we study the electric field-driven assembly of polystyrene (PS) microparticles located in the bulk of oil droplets, before being transported to droplet surfaces, and on drop surfaces. It has been reported that particle structuring and assembly depend on the type (DC or AC) and strength of the applied electric field, and the dielectric properties and electrical conductivity of the particles [9,21,22,23]. While it is simple to adjust the applied electric field, changing the properties of the particles is more challenging. Adjusting the electrical properties of PS particles is possible through sulfonation. Here, we investigate particle sulfonation as a method to produce PS particles with an increased dielectric constant and electric conductivity. This allows for different structuring and organization of PS particles at droplet interfaces. Particle sulfonation yields polymeric particles with a large range of electrical properties that are related to the degree of sulfonation, which can be controlled by several reaction conditions, including the reaction time, temperature, or concentration of sulfuric acid. Other physical properties may also change when PS particles are sulfonated for long periods of time, i.e., sulfonated PS particles become sticky, and this property can be used to create composite microcapsules with varied morphologies [24,25].

In this paper, we use electric fields to study the behavior of particles in the bulk of droplets and at droplet interfaces. The application of external electric fields has proven to be a flexible method for particle structuring and manipulation of droplets. When a weakly conductive droplet is subjected to a uniform electric field, charges build up at the interface of the droplet, resulting in electric stress that induces electrohydrodynamic (EHD) flows [16]. EHD flows can be used to indirectly structure particles at drop interfaces [23,26], for colloidal assembly [17], to fabricate patchy colloidal capsules [7], and to improve particle packing [27]. Particles can also be manipulated directly, by electric forces such as electrophoresis [28] or dielectrophoresis (DEP) [29,30,31], e.g., to separate particles at drop interfaces via gradients in the electric field [22] or to remove particles from drop surfaces using a tip streaming mechanism [32,33]. The structuring of particles can also occur via dipole-dipole interactions between particles, resulting in electrorheological chain formation [23,34,35] at drop interfaces.

In certain systems, both dielectrophoretic and EHD effects may be present, and depend on the applied electric field. By adjusting the frequency of the applied electric field, Amah and co-workers [21] demonstrated that the motion of particles can be switched between EHD flows transporting particles towards the drop equator, and DEP moving particles towards the drop poles. In our previous work, we discovered the “pupil effect”, where weakly conductive clay particles assemble at the drop equator and form a ribbon-like structure. The particle concentration and the ribbon width can be controlled by the electric field strength, i.e., at weak electric field strengths, EHD flows dominate and compress the ribbon, whereas at strong electric field strengths, particle dipolar interactions stretch the particle ribbon in direction towards the drop electric poles [23]. However, the effect was only achievable for particles with certain electrical properties: if the surface particles were too conductive, they only formed chain-like structures, while insulating particles only formed equatorial ribbons. This result motivated us to prepare a set of particles of the same size, shape, and material, but with different electrical properties. We have produced such a set of particles by sulfonating PS particles, which is an efficient, fast, and cheap method for modifying low-priced polystyrene particles.

The electrical properties of particles are also important in electrorheological (ER) fluids: colloidal or granular dispersions consisting of particles with a high dielectric constant or conductivity in an insulating (or weakly conductive) liquid. When subjected to external electric fields, the structure of the particles and the rheological properties of the ER fluids, such as the yield stress, apparent viscosity, storage, and loss moduli, may change drastically [36,37], allowing some ER fluids to change from a liquid to a gel and back, in time scales typically of the order of milliseconds. These characteristics make ER fluids useful in applications in microfluidic devices [38] and systems like hydraulic valves, clutches [39], brakes, and dampers [40,41]. In this work, we measure the flow of ER dispersions of modified PS particles in silicone oil, in response to applied electric fields at different strengths. We investigate how sulfonated PS particles change the electrorheological properties of PS suspensions, including the shear stress. The purpose of the ER experiments is not to pursue materials with high yield stresses, but rather to relate the electrorheological properties of PS suspensions with particle structuring inside droplets and at their interfaces. In addition, we perform Fourier transform infrared (FTIR) studies, measurements of particle dielectric constants and electrical conductance. These measurements complement and support the experiments on the electric field-driven assembly of particles. In short, all of the experiments demonstrate how sulfonation can be used to tailor electrical properties of PS particles and the importance of such properties in particle structuring in the bulk of droplets and at their interfaces.

## 2. Results

We use PS particles (mean diameter around 40 μm) sulfonated at incremental reaction times (2, 4, 8, 16, 32, 64, 128, and 256 min) to study the influence of the reaction time on the final physical properties of the sulfonated PS particles, and their electric field-driven assembly capabilities in an oil-in-oil system. The untreated PS particles, from which the sulfonated particles were made, are also studied here as a reference sample.

### 2.1. FTIR Studies

**Figure 1:**
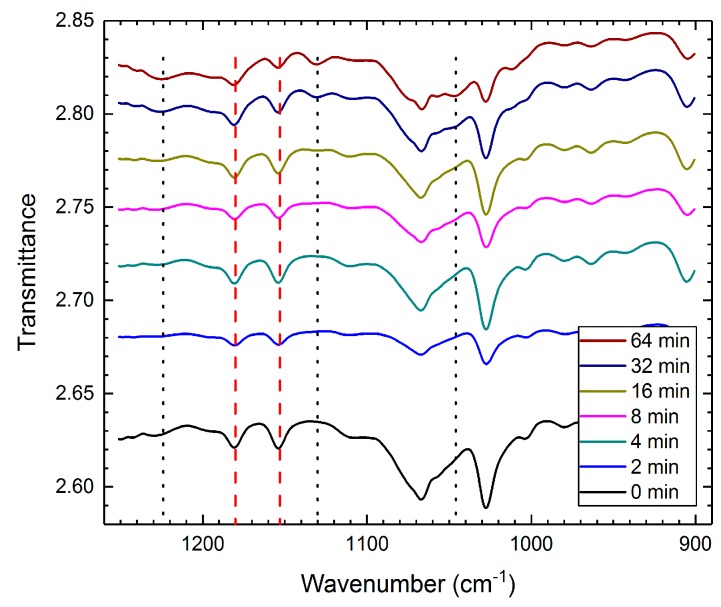
Stacked view of the FTIR transmission data. The line at 1046 cm^−1^ indicates the asymmetric stretching vibrations of the SO_3_^−^ groups, whereas the lines at 1180 and 1224 cm^−1^ indicate the symmetric stretching vibrations of the SO_3_^−^ groups. The appearance of a 1130 cm^−1^ band for the sulfonated polystyrene samples indicates the presence of sulfonate groups attached to phenyl rings. The red dashed lines indicate the bands used for calculating the transmittance ratio values.

To recognize the chemical changes of particles due to the sulfonation procedure, we collected FTIR spectra of the modified PS samples. Figure 1 shows a stacked view of the measured FTIR transmission data in the 900–1250 cm^−1^ region. The line at 1046 cm^−1^ indicates the asymmetric stretching vibrations of the SO_3_^−^ groups, whereas the lines at 1180 cm^−1^ and 1224 cm^−1^ indicate the symmetric stretching vibrations of the SO_3_^−^ groups [42,43]. The appearance of a 1130 cm^−1^ band for sulfonated polystyrene samples indicates the presence of sulfonate groups attached to phenyl rings, i.e., attributed to ring deformations [42,43,44]. Table 1 lists the calculated values of the transmittance ratios between the 1180 cm^−1^ and 1153 cm^−1^ bands (red dashed lines in Figure 1), where the latter band is either attributed to the C–H bending of the aromatic ring or the CH–CH_2_ stretching vibrations [45]. The increase of the transmittance ratio values with sulfonation time is correlated with the increased number of sulfonated groups. In short, the FTIR data consistently confirm that our sulfonation procedures impart sulfate groups to the surface of the PS particles.

### 2.2. Optical Photography and Electron Microscopy Examination of Sulfonated PS Particles

**Figure 2:**
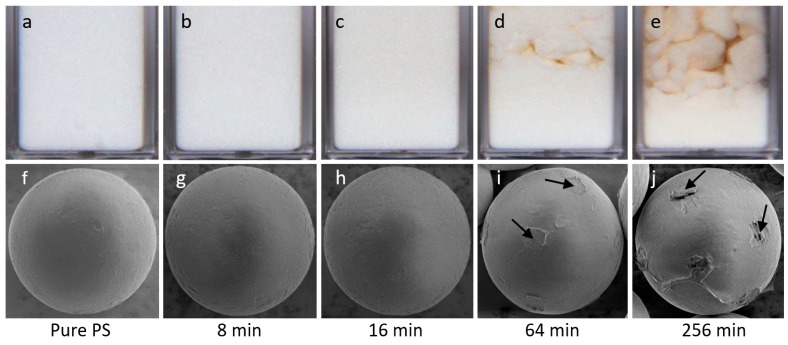
Optical photography (**a**–**e**) and scanning electron microscope (**f**–**j**) images of pure and sulfonated PS particles. The PS particles that were sulfonated for short periods of time remain white and powdery, resembling non-modified PS particles (**a**–**c**). When the PS particles are sulfonated for longer periods of time (64 and 256 min), they become yellowish, and during the washing and drying steps of particle sulfonation, they aggregate (**d**,**e**). Electron microscope images show that all the modified PS particles remain spherical and have sizes similar to non-modified PS particles (**f**–**j**). The PS particles modified for longer than around 1 h display inhomogeneities on their surfaces in the form of spots, some of them marked with black arrows (**i**,**j**). These spots are residuals of “necks” that were formed between fused PS spheres forming aggregated structures. During sample preparation for SEM imaging, the aggregated particles detach and the necks between the spheres break, leaving spots at the particle surfaces.

Optical photography and scanning electron microscope (SEM) images of both the pure and sulfonated PS particles are presented in Figure 2, in the top and bottom rows, respectively. Dry PS samples were placed in 10 mm × 10 mm plastic cuvettes and photographed. The PS particles sulfonated for short periods of time (up to around 32 min) resemble non-modified PS particles in appearance: they remain white and powdery (hard particles that do not adhere to one another) after the chemical treatment, see Figure 2a–c. The PS particles sulfonated for longer periods of time appear progressively more yellowish and tend to aggregate and form chunks, as shown in Figure 2d,e. They are also much more difficult to disperse in oil because they become more hydrophilic [46,47], aggregate, and adhere to one another. The yellow color is caused by the degradation of PS molecules at the surface of the microspheres. Previously, such a color change has been reported to increase with both the reaction time and the temperature [48,49].

SEM was used to study the surface morphology of the PS samples. The images in Figure 2f–j show that all of the modified PS particles remain spherical and have sizes similar to the untreated PS particles. The surface morphology of the particles sulfonated for 8 and 16 min look similar to that of the non-modified PS particles (Figure 2f–h). The particles sulfonated for around 1 h and longer, develop inhomogeneous surfaces in the form of rough spots—marked with black arrows in Figure 2i,j. The PS particles that are sulfonated for long periods of time start to fuse, form necks between each other, and eventually form aggregated structures. When such structures and interparticle necks break (for instance during sample preparation for SEM imaging), the detached particles are left with neck residuals. Adhesion between sulfonated particles has been observed by other researchers [24], and is generally expected for particles exposed to sulfonation for a long period of time [46].

For electro-rheological investigations and experiments focusing on electric field-driven particle assembly, we chose to use particles that were modified for no longer than 32 min, i.e., we used particles without significant morphological surface modifications. These particles disperse in oil more readily because they do not form large aggregates and are less hydrophilic than particles modified for longer periods of time.

### 2.3. Electrical Conductance and Dielectric Constant

To determine the electrical properties of the PS particles, including the dielectric constant (*ε*) and electrical conductivity (*σ*), we carried out capacitance measurements using an LCR meter at a frequency (*f*) of 100 Hz. The LCR meter was set to capacitor mode, where the capacitance (*C*) and the dissipation factor (tan *δ*) were recorded. The dielectric constant and electrical conductance (*G*) values were calculated using equation: *ε* = *C*/*C*_o_, *G* = 2π*fC*tan *δ* (for details see Section 4). The conductance was then converted to conductivity. Table 2 lists the calculated values of *ε* and *σ*. The dielectric constant increases with the sulfonation reaction time, starting from the sample sulfonated for 4 min and reaching a maximum value for the PS sample modified for 32 min, which is one order of magnitude higher than that of pure PS particles. A similar trend applies to the measured conductivity values. The highest electrical conductivity value is obtained for the sample sulfonated for 32 min, and is three orders of magnitude higher than that of the pure PS particles. This is expected because the degree of sulfonation increases with the reaction time [50], allowing more water to be absorbed on the PS samples [25,51], thus enhancing the protonic conductivity [52]. To test this assumption, we dried the sulfonated PS particles by lyophilisation and measured the electrical properties of the lyophilized samples. The results of these measurements are included in Table 2. Both the dielectric constant and electrical conductivity values of all the lyophilized PS sulfonated particles are significantly smaller than those of the initial PS sulfonated samples, but are still larger than non-modified PS particles. This indicates that water content plays an important role in the electrical conductivity and polarization of the sulfonated PS particles. Previously, such water dependency on the dielectric properties of sulfonated PS films has been reported [53]. To produce a broad distribution of particles in terms of the electrical properties, we decided to use the initial sulfonated PS particles (not lyophilized) for further experiments on electro-rheology and electric field-driven particle assembly.

### 2.4. Electro-Rheology

**Figure 3:**
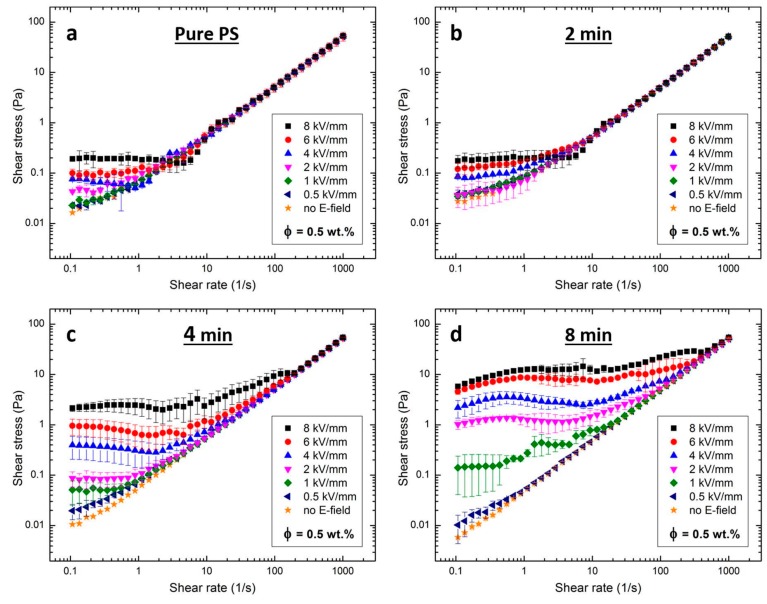
Log-log plots of flow curves of silicone oil dispersions with non-modified PS particles (**a**); and with PS particles sulfonated for 2 min (**b**); 4 min (**c**); and 8 min (**d**). The silicone oil dispersions are subjected to electric field strengths between 0.5 and 8 kV/mm, and a particle concentration of ~0.5% by weight was used to prepare all the silicone oil (50 cSt) dispersions.

Figure 3a–d presents the flow curves for silicone oil dispersion with non-modified PS particles, and three dispersions with PS particles sulfonated for 2, 4, and 8 min, respectively. The particle concentration in all dispersions is 0.5% by weight, similar to the particle concentration in silicone oil dispersions utilized for the electric field-driven assembly experiments, described in the next section.

When no electric field is applied, the dispersions behave as Newtonian fluids, i.e., their shear stress values increase linearly with the shear rate (remark: Two electrical grounding brushes connected to the measuring bob may induce an artificial yield stress of up to 0.05 Pa). At low shear rates, we observe small non-Newtonian behavior (nonlinear), as seen in Figure 3a,b. The dispersions behave as Bingham fluids when subjected to DC electric fields of strengths 0.5, 1, 2, 4, 6, and 8 kV/mm, i.e., a viscoelastic material that acts as a rigid body when subjected to low stresses and flows as a viscous fluid when subjected to high stresses [54]. This behavior is particularly apparent for dispersions in strong electric fields.

Table 3 lists the shear stress values of dispersions measured at a shear rate equal to 0.1 s^−1^ and subjected to different electric field strengths. At the strongest electric field strength (8 kV/mm), the PS particles sulfonated for 8 min exhibit the largest electrorheological response, and the yield stress value is nearly 30 times higher than that observed for the pure PS particle dispersion. The values of the shear stress generally increase with applied electric field strength and sulfonation time. For electrorheological fluids, this behavior is usually attributed to either differences in dielectric properties, and/or conductivities between the particles and carrier liquid, and simple polarization or conduction models apply [55,56,57]. The electrorheological results presented here are consistent with measurements of the dielectric constant and electrical conductance shown in the previous section.

### 2.5. Electric Field-Driven Particle Assembly

We use electric fields to manipulate particles both inside the droplet and at the droplet surfaces. The particles are set in motion and assemble either by: (i) direct interaction of the applied electric field, i.e., through electrophoresis and/or dielectrophoresis; or (ii) indirectly, through circulating liquid flows induced by DC or low frequency AC electric fields. Here, we qualitatively demonstrate how the dipolar interaction force between modified particles changes with the particle sulfonation reaction time. We show interesting interactions between viscous forces (induced by EHD flows) and dipolar forces, creating different particle assemblies.

**Figure 4:**
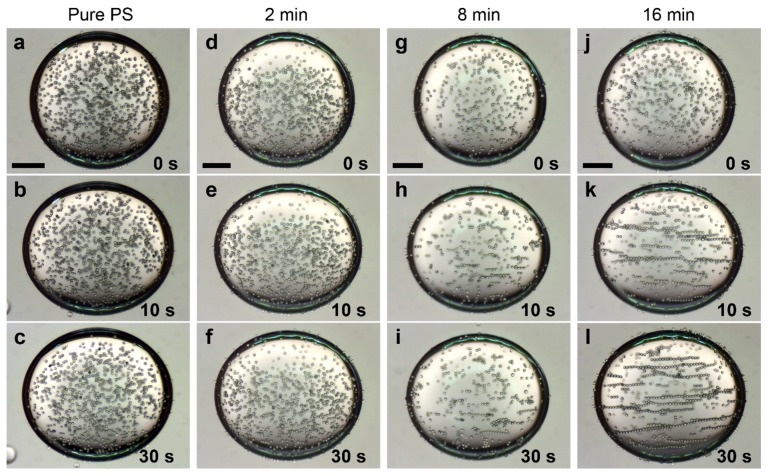
Four silicone oil droplets suspended in castor oil, each containing different particles, from left to right column: non-modified PS particles, and PS particles sulfonated for 2, 8, and 16 min. Initially, the particles in each droplet are randomly dispersed, and the majority of the particles are in the droplet bulk (**a**,**d**,**g**,**j**). The electric field is zero at *t* = 0 s (first row), then a 100 Hz square wave electric field of strength 600 V/mm is applied (rows 2 and 3). The non-modified PS particles do not experience any significant motion after the application of the electric field (**b**,**c**). The same applies for the PS particles sulfonated for 2 min (**e**,**f**). The PS particles sulfonated for 8 min clearly interact with one another, and short chains are present 10 s after the application of the electric field (**h**,**i**). Dipolar chaining is most distinct for the PS particle sulfonated for 16 min (**k**,**l**). Scale bars are 500 μm.

We apply AC electric fields to observe whether (and how fast) the PS particles form dipolar chains. This gives an indication of the dipolar forces acting on the different PS particles. The electric field frequency was set to 100 Hz, a value far above the critical frequency at which EHD liquid flows occur in this system (few Hz) [23]. Four silicone oil droplets with radii of around 1 mm were suspended in castor oil, each containing particles with different degrees of sulfonation, including non-modified PS particles and PS particles sulfonated for 2, 8, and 16 min. Initially, the particles in each droplet are randomly dispersed, and the majority of them are located in the bulk of a droplet (Figure 4a,d,g,j). Each droplet is subjected to an AC electric field of 600 V/mm. The non-modified PS particles (Figure 4b,c) and the PS particles sulfonated for 2 min (Figure 4e,f) do not experience any significant motion within the time frame of the experiment. Only a few short particle chains are observed, approximately aligned in the direction of the electric field lines. The PS particles sulfonated for 8 min clearly interact with one another, forming short chains visible 10 s after the application of the electric field (Figure 4h,i). The highest number of dipolar chains are formed for the dispersion containing PS particle sulfonated for 16 min. The interactions between these particles are very strong, and the long chains are formed in just a few seconds (Figure 4k,l). Such particle behavior is consistent with the results presented in previous Section 2.2 and Section 2.3. For the sake of image clarity, we did not include the experiment for droplets with samples sulfonated for 4 min (particle behavior is qualitatively similar to that of the sample modified for 2 min) and for 32 min (particle behavior is qualitatively similar to that of the sample modified for 16 min).

**Figure 5:**
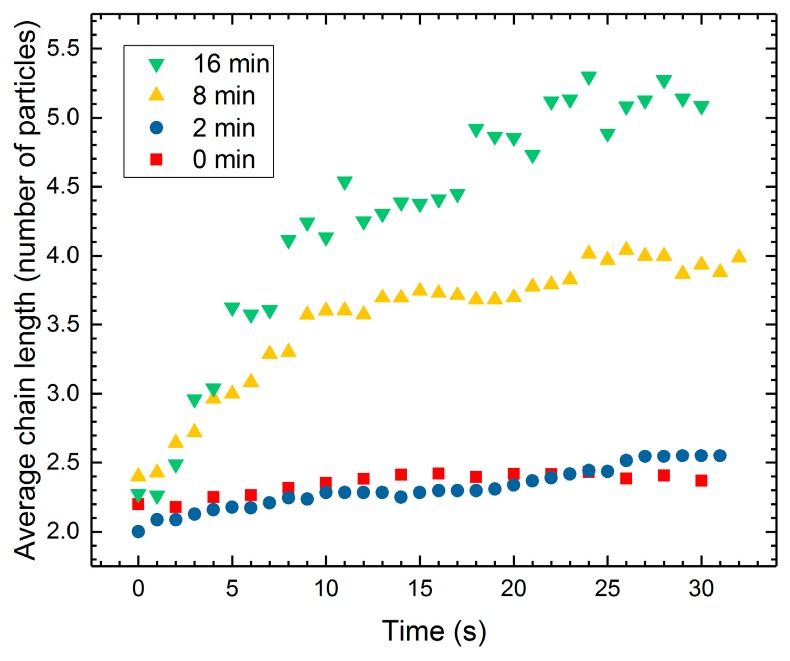
Average length of chains plotted against time for PS particles used in the four droplets presented in Figure 4.

In Figure 5, we plot the average chain length versus time for the four droplets with different PS particles (presented in Figure 4). In the absence of an electric field, the average chain length is around two particles for the chains present in all four droplets. After the electric field is applied, the PS particles interact via dipolar interactions and the length of the chains increases. The pure PS particles (red squares) and PS particles sulfonated for 2 min (blue circles) form short chains (average chain length after 30 s is around 2.5 particles), while the PS particles sulfonated for 8 min (yellow triangles) and 16 min (green down pointing triangles) form longer chains, and the average chain length after 30 s is around four to five particles, respectively. Quantitatively, this consistently shows that the dipolar force between the PS particles increases with sulfonation time, allowing particles to form longer chains in the droplet. We attribute this result to the increased electrical properties of the particles obtained through sulfonation (see Table 2). For AC electric fields, the dipolar force between two particles is proportional to the complex particle polarizability [58]: $Q^{2}=\left(\sigma_{p}^{*}-\sigma_{\mathrm{in}}^{*}\right)/\left(\sigma_{p}^{*}+2\sigma_{\mathrm{in}}^{*}\right)$, where $\sigma_{p}^{*}=\sigma_{p}+i\omega \varepsilon_{0}\varepsilon_{p}$ and $\sigma_{\mathrm{in}}^{*}=\sigma_{\mathrm{in}}+i\omega \varepsilon_{0}\varepsilon_{\mathrm{in}}$ are the complex conductivities, $\sigma_{p}$ and $\sigma_{\mathrm{in}}$ are the conductivities of the particles and droplet, ω is the angular frequency of the electric field, $\varepsilon_{0}$ is the vacuum permittivity, and $\varepsilon_{p}$ and $\varepsilon_{\mathrm{in}}$ are the dielectric constant of the particles and the droplet. If we insert numbers and compare the $Q^{2}$ values of the non-modified PS particles (0 min) and particles sulfonated for the longest time (32 min), we obtain: $Q_{32}^{2}/Q_{0}^{2}\approx 2.3$. This calculation shows that the increased electrical properties of the PS particles (through sulfonation) can double the dielectric force, supporting our observations.

**Figure 6:**
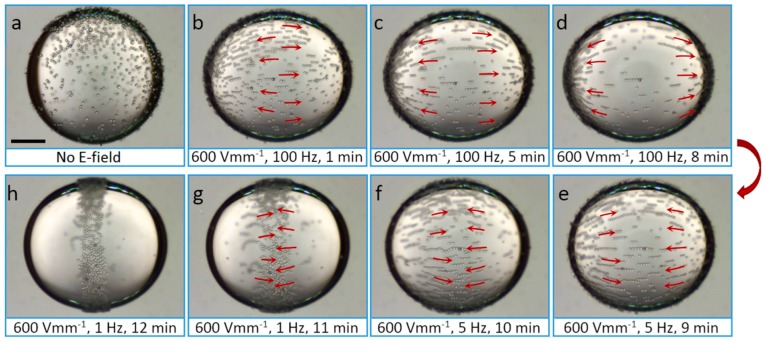
A silicone oil droplet containing pure PS particles and suspended in castor oil. The majority of particles are at the interface, randomly dispersed (**a**). An electric field of strength 600 V/mm is applied through the whole experiment, and the frequency is adjusted between 100 Hz (**b**–**d**), 5 Hz (**e**,**f**), and 1 Hz (**g**,**h**). By tuning the frequency of the electric field, it is possible to change the surface particle arrangements. Scale bar is 500 µm, and the droplet radius around 1 mm.

**Figure 7:**
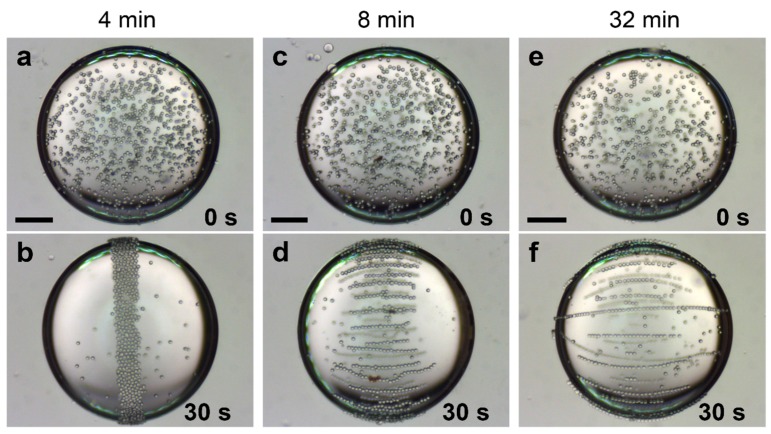
Three silicone oil droplets suspended in castor oil, each covered by particles with different degrees of sulfonation, including PS particles sulfonated for 4, 8, and 32 min. The majority of particles are at the interface, randomly dispersed (**a**,**c**,**e**). Each droplet is subjected to a DC electric field of 150 V/mm, initiating particle movement. The PS particles sulfonated for 4 min are carried by circulating EHD flows towards the electric equator and assemble into a “ribbon-like” structure (**b**). For the PS particles sulfonated for the longest time, i.e., with the largest dielectric constant and electrical conductance, we observe particle chain-structures that align along the electric field lines and span the entire droplet (**f**). Interesting particle organization takes place for the PS particles modified for 8 min. Due to the combined action of EHD flows and dipolar attractive interactions between the particles, chain like assemblies form and are structured at the electric equator (**d**). Scale bars are 500 μm and the droplet radii are around 1 mm.

As mentioned above and demonstrated in Figure 4, we apply AC electric fields with a frequency of 100 Hz to prevent the induction of EHD flows. We observe similar particle chaining effects, as presented in Figure 4, when we apply AC electric fields with higher frequencies, i.e., up to around 5 kHz, which is the limit of our voltage amplifier. However, if we decrease the frequency of the electric field, different phenomena take place. Figure 6 shows a silicone oil droplet with pure PS particles adsorbed at the interface. The particles were brought to the droplet interface and then gently dispersed by mechanical stirring using a pipette tip. As a result, the majority of the particles are initially randomly dispersed at the droplet interface, before the application of an electric field (Figure 6a). When an electric field (600 V/mm) with a frequency of 100 Hz is applied, all of the particles are guided towards the electric poles through the action of DEP (Figure 6b–d) [21]. The direction of the DEP force is determined by the sign of $\beta \overset{´}{\beta }=Re\left(\frac{\varepsilon_{\mathrm{in}}^{*}-\varepsilon_{\mathrm{ex}}^{*}}{\varepsilon_{\mathrm{in}}^{*}+2\varepsilon_{\mathrm{ex}}^{*}}\right)Re\left(\frac{\varepsilon_{p}^{*}-\varepsilon_{\mathrm{ex}}^{*}}{\varepsilon_{p}^{*}+2\varepsilon_{\mathrm{ex}}^{*}}\right)$, the Clausius-Mossotti factor of the droplet and particle with respect to the exterior fluid [21]. The complex permittivity is $\varepsilon^{*}=\varepsilon -i\sigma /\omega$, where ω is the angular frequency of the applied electric field. If $\beta \overset{´}{\beta }<0$, the particles aggregate at the equator, and if $\beta \overset{´}{\beta }>0$, the particles aggregate at the droplet poles. Inserting numbers yields $\beta \overset{´}{\beta }\approx 0.05$ for the studied silicone-castor oil system (with pure PS particles), which is consistent with the DEP force direction observed in our experiments. The interparticle dipolar forces are sufficiently strong (compared to the DEP force) for the PS particles to form chains. Note that the dipolar interaction of particles at the droplet interface is stronger than when they are in the bulk of the droplet, and even non-modified PS particles form chains in the presence of an AC electric field. After we decrease the frequency of the electric field to 5 Hz, the particles are transported in the opposite direction, i.e., towards the electric equator (Figure 6e,f). This is due to induced EHD flows, which arise when free charges accumulate on the interface of the droplet [16,59,60]. In this silicone oil droplet in castor oil system, the direction of these flows is directed from the electric poles to the electric equator of the droplet. Experimentally, we observe that the drag force on a particle due to the EHD flows dominates the DEP force for frequencies below ~10 Hz. The EHD drag force increases with a decreasing frequency, as 1/*ω*^2^ [21]. At 5 Hz, the EHD flows are weak because the charges do not have sufficient time to accumulate at the droplet interface (for a silicone oil droplet in castor oil system, the time for the interface to acquire its steady-surface charge distribution is around 1 s). If we decrease the frequency to 1 Hz, the particles move towards the electric equator more rapidly (Figure 6g,h), where they stay and form an equatorial ribbon-like structure (Figure 6h). At this frequency, the EHD drag force dominates both the DEP and dipolar force. As a result, the particle chains made of non-modified PS particles are broken into smaller chains or single particles. When applying AC electric fields with low frequencies or DC fields, the modified PS particles may form a compacted ribbon-like structure, or short dipolar chains assembled at the electric equator of a droplet, or make long dipolar chains. The particle organization depends on the dielectric properties and electric conductivity of the PS particles (presented in Figure 7).

Figure 7 shows three silicone oil droplets suspended in castor oil (same oils and droplet sizes as used in Figure 4), each covered by particles with different degrees of sulfonation, including PS particles sulfonated for 4, 8, and 32 min. The particles were brought to the interface and then gently dispersed by mechanical stirring using a pipette tip. As a result, a majority of the particles are randomly dispersed at the droplet interface (Figure 7a,c,e). In this system, the particle density is similar to those of both oils, so the gravitational force (vertical direction in all images) can be neglected. Each droplet is subjected to a DC electric field, initiating particle movement. We use an electric field strength of 150 V/mm, not 600 V/mm as in the experiments with AC fields, because the EHD flows that are present in DC electric fields generate instabilities on the droplet surface, which may lead to the formation of spinning domains [23,61], as presented in Figure S1.

In addition to the gravitational force, the PS particles at the droplet interface experience three different forces when subjected to a DC electric field: EHD drag, DEP and dipolar forces. In Section 2.3, we ascertained that the electrical properties of the PS particles increase with the sulfonated time. For the particles sulfonated for short periods of time, the low dielectric constant and electric conductivity (Table 2) yield DEP and dipolar forces that are ~2–3 orders of magnitude smaller than the EHD drag force. Consequently, EHD circulation flows govern the dynamics of PS particles sulfonated for short periods of time (4 min), i.e., the particles follow the EHD flows and are brought to the droplet equator where they form a ribbon-like structure (Figure 7b) [23,62]. For these particles, we do not observe any direct electrical interactions (e.g., electrophoretic motion or dipolar interaction between the particles). A similar behavior is observed for pure PS particles and PS particles sulfonated for 2 min (not shown here). The direction of the EHD flows and the droplet deformation are determined by the ratios between the conductivities and dielectric constant of the fluids [23,63,64]. In our case, the surrounding castor oil conducts approximately 10 times better than the silicone oil, thus resulting in an oblate deformation (droplet axis perpendicular to the electric field direction is larger than the axis parallel to the electric field direction), and the EHD flows are directed from droplet electric poles to the electric equator [16]. The PS particles sulfonated for longer periods of time have higher dielectric constants and conductivities (than the PS particles sulfonated for shorter times), resulting in stronger dipolar interactions.

For DC electric fields, the dipolar force between two particles is proportional to the factor [65]: $\beta^{2}={\left(\sigma_{p}-\sigma_{\mathrm{in}}\right)}^{2}/{\left(\sigma_{p}+2\sigma_{\mathrm{in}}\right)}^{2}$, involving the conductivities of the particles $\sigma_{p}$ and the surrounding fluid $\sigma_{\mathrm{in}}$. If we insert numbers and compare β between the non-modified PS particles (0 min) and the particles sulfonated for the longest time (32 min), we get: $\beta_{32}^{2}/\beta_{0}^{2}\approx 360$. This shows that the sulfonation process results in larger interparticle dipolar forces. Particles are able to form chains when the dipolar force exceeds the EHD drag force. This is the case for the PS particles modified for 8 min, where the combined action of the EHD flows and dipolar forces between the particles leads to chain-like assemblies being formed and structured at the electric equator of the droplet (Figure 7d). The dipolar force between the particles is attractive when they are aligned parallel to the electric field direction, and repulsive when aligned perpendicular to the electric field direction. That is why the particle chains are separated. 

If the dipolar force is sufficiently strong compared to the EHD drag force, the particles can form chains from droplet pole to droplet pole (Figure 7f), and suppress or remove the EHD flows by short cutting the electric poles [23]. These chains conduct electric current much better than oils and form electrical short cuts between the two droplet electric poles. As a result, the droplet becomes less oblate (Figure 7f) and can even stretch and acquire a prolate shape if the particle concentration and electric field are higher. 

The DEP force on the particles also increases with the dielectric constant and electric conductivity of the particles. However, the increase in the DEP force between non-modified particles (0 min) and particles sulfonated for a long time (32 min) is only one order of magnitude (for equations see [21]). The DEP force is thus dominated by both EHD and dipolar forces, and it is safe to neglect DEP forces in this experiment.

## 3. Discussion and Conclusions

In this work, we have investigated how sulfonation changes the electrical properties of PS particles and how this modification affects particle organization at the interfaces and bulk of droplets subjected to electric fields. To study how our sulfonation procedure changes the properties of the PS particles, the particles were sulfonated for different lengths of time and compared. FTIR studies of the sulfonated PS particles show that our sulfonation procedures impart sulfate groups to the surface of the PS particles. 

We measured the electric conductivity and dielectric constant of pure and modified PS particles. The measurements consistently show that these electric parameters increase with sulfonation time. Electro-rheology experiments were also performed on pure and sulfonated PS particles dispersed in silicone oil. Consistent with the measured electrical properties, we find that the shear stress value of the particle dispersions increases with the sulfonation reaction time. We can thus conclude that our sulfonation procedure brings sulfate groups to the surface of the PS particles, and that the degree of sulfonation increases the dielectric constants and electric conductivities) of the PS particles.

We also studied how these modified particles organize in the bulk and interfaces of droplets, when subjected to electric fields. PS particles with different degrees of sulfonation were placed in silicone oil droplets and suspended in castor oil. First, we applied AC electric fields to study the formation of particle chains and structuring of particles at droplet poles. The electric field strength, frequency, and fluids were kept constant, with particle properties being the only variable parameters. We observed that the PS particles sulfonated for long periods of time (32 min) form longer chains compared to PS particles sulfonated for shorter periods of time (0 min). This is attributed to the change in the electrical properties, affecting the dipolar force between particles. Next, we studied how PS particle organization changes with the frequency of the applied electric field. When we apply an electric field with a high frequency (100 Hz), the PS particles form chains and move towards the droplet poles. This movement is caused by DEP forces. When we decrease the frequency (to 5 and 1 Hz), the particles move in the opposite direction, towards the droplet equator. This is because free charges have time to accumulate at the droplet interface, inducing EHD flows with a direction from the droplet poles to the droplet equator. The EHD drag force experienced by the particles increases with the electric angular frequency as 1/*ω*^2^, and becomes stronger than both the DEP and dipolar forces when the electric field frequency is sufficiently low. Particles chains are then broken into smaller chains or individual particles, and assemble in a ribbon at the droplet equator.

In the last part of the paper, we utilized DC electric fields. With particle properties as the only variables, we observed that PS particles sulfonated for longer periods of time form long chains, while PS particles sulfonated for shorter periods of time do not form chains at all. Our calculations show that the dipolar force between particles can increase by three orders of magnitude when we compare pure PS particles with PS particles sulfonated for 32 min. The dipolar force is weaker than the EHD drag force for the pure PS particles, preventing them for forming chains. When PS particles are sulfonated, the dielectric constant and electric conductivities increase, resulting in dipolar forces that exceed the EHD drag force.

In the future, we plan to extend these studies to mixtures of particles with different electrical properties, and also vary both the strengths and frequencies of the electric fields. We foresee that by doing so, it will be possible to obtain new particle architectures at droplets surfaces. PS particles can be easily sintered and this allows us to permanently lock the assembled structure formed, for example, Janus shells with anisotropic properties.

We note that the PS particles in the bulk of the droplet arrive at the droplet interface much faster than particles of the same size made of polyethylene particles that we studied before [23]. This is possibly due to charges that sulfonated particles acquire. It was demonstrated that the values of the zeta potential increase as the sulfonation reaction progresses to up to few hours [24].

As a final remark, we discuss the importance of the wettability of PS particles. Once the sulfonated PS particles are brought to a drop surface, they bind to it with a binding energy proportional to $a^{2}\gamma {\left(1\pm \cos\theta \right)}^{2}$, where a is the particle radius, *γ* is the surface tension between the drop and surrounding fluids, and θ is the contact angle. The sign inside the bracket is defined as positive for the removal of particles into the outer surrounding liquid. The particle binding energy to the interface is maximum when the contact angle is equal to 90°. Generally, PS particles with a high degree of sulfonation become more hydrophilic than non-modified PS particles, and thus have a higher affinity towards castor oil than silicone oil. Experimentally, we observe that the sulfonated PS particles bind weakly to droplet interfaces and may detach due to strong EHD flows or dipolar interactions, as presented in Figure S2.

## 4. Materials and Methods

### 4.1. Sulfonation of PS Particles

The PS particles were purchased from Microbeads AS, Skedsmokorset, Norway (Dynoseeds TS40 6317) with mean diameters of around 40 μm, a density of around 1.05 g·cm^−3^, and without cross-linking. The general preparation route for the chemical modification of PS particles was as follows. Firstly, sulfuric acid (5 mL, 98%, 231-639-5, Chempur, Piekary Śląskie, Poland) was poured into a glass vial filled with polystyrene powder (5 g). During the chemical reaction, the temperature was maintained at 50 °C by placing the vial into a water bath. The powder dispersion was continuously stirred using a magnetic stirrer. After the respective stirring time, the dispersion was neutralized using a concentrated potassium hydroxide water solution. Subsequently, the dispersion was poured into a sintered glass funnel (connected to a side-arm flask with a tube leading to a vacuum pump), and the sulfonated polystyrene particles were rinsed thoroughly with distilled water. Finally, the sulfonated particles were dried at 75 °C for 24 h. To study the influence of the reaction time on the final physical properties of the sulfonated particles, we made eight samples prepared at incremental reaction times: 2, 4, 8, 16, 32, 64, 128, 256 min, respectively. The schematic representation of the sulfonation procedure is presented in Figure S3. We studied the influence of water content on the electrical properties of sulfonated PS particles by lyophilization conducted in a freeze dryer (IlshinEurope TFD5503) at −50 °C and 50 mTorr for 24 h. Before the lyophilization, the samples were stored in a freezer at −20 °C for 24 h.

### 4.2. FTIR Studies

A Tensor 27 FTIR spectrometer (Bruker Optics, Ettlingen, Germany) equipped with a single reflection diamond ATR unit was utilized for the IR analyses. Before each spectral acquisition session, the background was recorded and the background spectra were subtracted from the FTIR spectrum of each sample. The FTIR-ATR spectra were then recorded in the 900–1250 cm^−1^ range by running 512 scans with a resolution of 2 cm^−1^.

### 4.3. Optical Photography and Scanning Electron Microscopy Imaging 

The dry samples in Figure 2 (top row) were photographed using a digital camera (Canon EOS 700D, Tokyo, Japan). The structural surface characterization of the sulfonated PS particles presented in Figure 2 (bottom row) was performed by scanning electron microscopy, using a JEOL JSM-7001F TTLS (JEOL Ltd., Tokyo, Japan). We prepared the powder samples by spreading a thin layer of the powder onto a double-sided conductive carbon tape. The surface of the uncoated samples was imaged via secondary electron imaging and observed at an accelerating voltage of 1 kV, to avoid a charging effect.

### 4.4. Measurements of Dielectric Constant and Electric Conductivity

To determine the dielectric constant and the electrical conductance of the powder samples, we carried out capacitance measurements by means of an LCR meter (Agilent E4980A, Santa Clara, CA, USA) at a frequency (*f*) of 100 Hz. A home-made cylindrical capacitor (with dimensions depicted in Figure S4) was used as a sample holder, where the separation distance between the inner and outer cylindrical electrode was 1.4 mm, which gives a capacitance value of the empty capacitor (*C*_o_) equal to 2.78 pF. The studied sample was poured into the cylindrical gap, while the homogenous filling was provided by shaking the capacitor. We performed measurements with an effective voltage value of 1 V at room temperature (24 °C) and an ambient pressure. The LCR meter was set to parallel mode, where the capacity (*C*) and the dissipation factor (tan *δ*) were recorded. Then, the dielectric constant (*ε*) and conductance (*G*) values were calculated by: *ε* = *C*/*C*_o_, *G* = 2π*fC*tan *δ*. Finally, the conductance was converted to the electrical conductivity (*σ*).

### 4.5. Rheometry

The electro-rheological properties of the sulfonated PS particle dispersions were measured under direct current (DC) electric fields using a Physica MCR300 Rotational Rheometer equipped with a coaxial cylinder Physica ERD CC/27 (Malvern Instruments Ltd., Malvern, UK). Silicone oil (Dow Corning, Auburn, AL, USA) with a viscosity of 50 cSt, an electrical conductivity of approximately 3–5 pS·m^−1^, and a relative permittivity of around 2.8, was used for the experiments. All the rheological measurements were performed at a constant temperature of 23 °C. The flow curves were collected in the shear rate range between 0.1 and 1000 s^−1^, and at different electric field strengths, namely 0.5, 1, 2, 4, 6, and 8 kV/mm, respectively.

### 4.6. Experimental Set-Up for Electric Field-Driven Particle Assembly

The experimental set-up for the electric field-driven particle assembly of PS particles consisted of a sample cell placed on a mechanical x-y-z translational stage, a digital microscope, a signal generator, a voltage amplifier, an oscilloscope for monitoring signal shape and amplitude, and a PC for recording images. We used 10 mm × 10 mm plastic cuvettes (typically used for light spectroscopy) with two copper plates constituting electrodes, as a sample cell. Castor oil (83912, Sigma-Aldrich, St. Louis, MO, USA, density of 0.961 g·cm^−3^ at 25 °C, electrical conductivity of around 60 pS·m^−1^, relative permittivity 4.7, and viscosity of around 700 cSt) was poured into the cell. Silicone oil droplets containing PS particles were introduced into the castor oil by a mechanical pipette. To minimize the buoyancy force on the droplet with particles, two silicone oils (200/10 cSt and 200/100 cSt, Dow Corning, Auburn, AL, USA, electrical conductivity approximately 3–5 pS·m^−1^, relative permittivity 2.8) with densities of 0.960 and 0.965 g·cm^−3^ (measured at 25 °C) were adequately mixed, to match the castor oil density. The AC electric signal was always square-shaped and bipolar, and its RMS value (i.e., half of the peak-to-peak value) was provided in the text and figure captions. Optical microscopy observations of the electric field-driven particle assembly of PS particles presented in Figure 4, Figure 6 and Figure 7, were performed using a CMOS camera (UI-3590CP-C-HQ, IDS Imaging Development Systems GmbH, Obersulm, Germany) mounted on a high-magnification zoom lens system (MVL12X3Z, Thorlabs, Inc., Newton, NJ, USA). The experimental set-up is presented in Figure S5. All droplets have similar radii (~1 mm), and the small differences in their size do not affect the observed effects of particle structuring.

## Figures and Tables

**Table 1:** Lists of the calculated values of the transmittance ratios between the bands at 1180 cm^−1^ and 1153 cm^−1^. The increase of the transmittance ratio values with sulfonation time is correlated with the increased number of the sulfonated groups imparted to the surface of the PS particles.

Reaction Time (min)	0	2	4	8	16	32	64
Transmittance Ratio	1	1	1	1.001	1.002	1.007	1.009

**Table 2:** Lists of the calculated electrical values, including the particle dielectric constant (*ε*) and electrical conductivity (*σ*) measured at a frequency of 100 Hz.

Reaction Time (min)	0	2	4	8	16	32
Dielectric constant, *ε*	1.5	1.4	2.2	4.5	8.1	19.5
Dielectric constant, *ε* (lyophilized)	1.3	1.3	1.4	1.4	1.8	3.0
Electrical conductivity, *σ* (nS/m)	0.08	3.9	9.3	31.0	95.9	107.5
Electrical conductivity, *σ* (nS/m) (lyophilized)	0.08	0.8	1.6	1.6	8.5	30.1

**Table 3:** Shear stress values (*τ*) measured at shear rate 0.1 s^−1^.

Silicone Oil Dispersions with:	E (kV/mm)	0.5	1	2	4	6	8
pure PS particles	*τ* (Pa)	~0.02	~0.02	~0.04	~0.07	~0.10	~0.19
PS particles sulfonated for 2 min	*τ* (Pa)	~0.03	~0.03	~0.03	~0.08	~0.11	~0.18
PS particles sulfonated for 4 min	*τ* (Pa)	~0.02	~0.05	~0.08	~0.40	~1.00	~2.00
PS particles sulfonated for 8 min	*τ* (Pa)	~0.01	~0.13	~1.00	~2.00	~4.00	~5.50

## Supplementary Materials

The following are available online at www.mdpi.com/1996-1944/10/4/329/s1. Figure S1: Instabilities on droplet interface at high DC electric fields, Figure S2: Particle detachment from a silicone oil droplet suspended in castor oil, Figure S3: Schematic illustration of the sulfonation process of spherical PS particles, Figure S4: A home-made cylindrical capacitor used as a sample holder for measuring electrical capacitance and dielectric constants, Figure S5: Experimental set-up for the electric field-driven particle assembly of PS particles consisted of a CMOS camera.
