# Supplementary material for: Electric Field-Driven Assembly of Sulfonated Polystyrene Microspheres
Source: Materials (Basel). 2017 Mar 23;10(4):329. doi: 10.3390/ma10040329 (PMC5507009; doi:10.3390/ma10040329)
Supplement: Supplementary file 1 [file materials-10-00329-s001.pdf]

Supplementary Materials: Electric Field-Driven Assembly of Sulfonated Polystyrene Microspheres

Alexander Mikkelsen [1], Jarosław Wojciechowski [2], Michal Rajňák [3,4], Juraj Kurimský [4], Khobaib Khobaib [1], Ahmet Kertmen [5] and Zbigniew Rozynek [1,*]

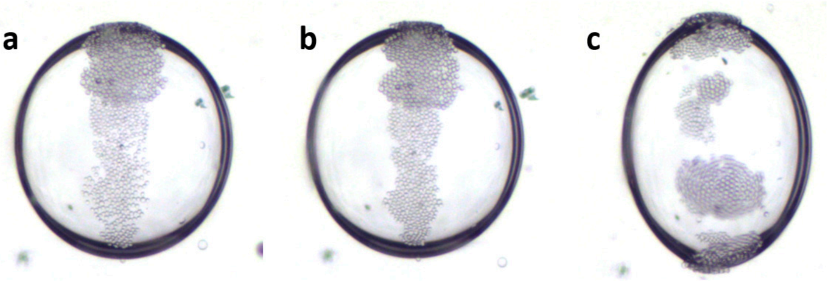

Figure S1. Instabilities on droplet interface at high DC electric fields. A silicone oil droplet containing pure PS particles (diameter 40 μm) and suspended in castor oil. The particles are at the droplet interface. A DC electric field of strength 100 V/mm (**a**), 200 V/mm (**b**) and 600 V/mm (**c**) is applied in horizontal direction. When a strong electric field is applied, instabilities develop at the droplet interface, leading to the formation of spinning particle domains (**c**). The droplet radius is around 1 mm.

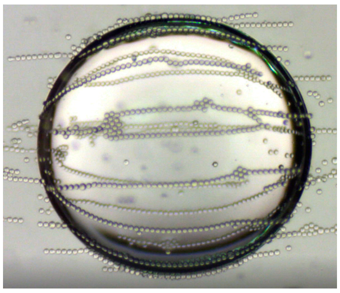

Figure S2. Particle detachment from a silicone oil droplet suspended in castor oil. The droplet is covered with 40 μm PS particles sulfonated for 32 min. An AC electric field (100 Hz) of strength 400 V/mm is applied in horizontal direction. The dipolar interactions between particles forming chains are strong, and some of the particles eventually detach from the droplet interface. This is because the particles have high affinity to the surrounding castor oil, resulting in low binding energy to the drop interface.

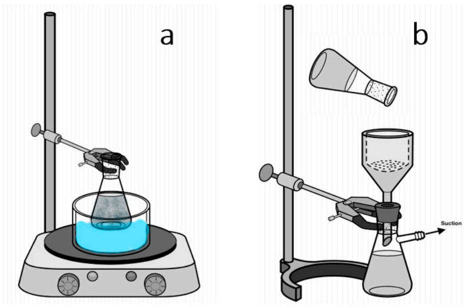

Figure S3. Schematic illustration of the sulfonation process of spherical PS particles. PS particles were placed in a small Erlenmeyer flask with sulphuric acid at 50 °C (placed in a water bath) and stirred using a magnetic stirrer (**a**). After the specific reaction periods, sulfonated polystyrene particles were filtrated by suction through a filter (**b**). Once the sulphuric acid was filtered out, the particles were washed with deionized water, saturated in KOH solution and then washed with deionized water again. Finally, the PH value was measured to control that the particles were properly neutralized.

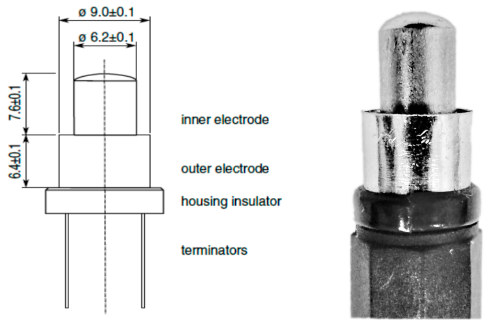

Figure S4. A home-made cylindrical capacitor used as a sample holder for measuring electrical capacitance and dielectric constants. The separation distance between the inner and outer cylindrical electrode was 1.4 mm, which gives a capacity value of the empty capacitor (C_o) equal to 2.78 pF. PS samples were poured into the cylindrical gap, while a homogenous filling of the cell was ensured by properly shaking of the capacitor.

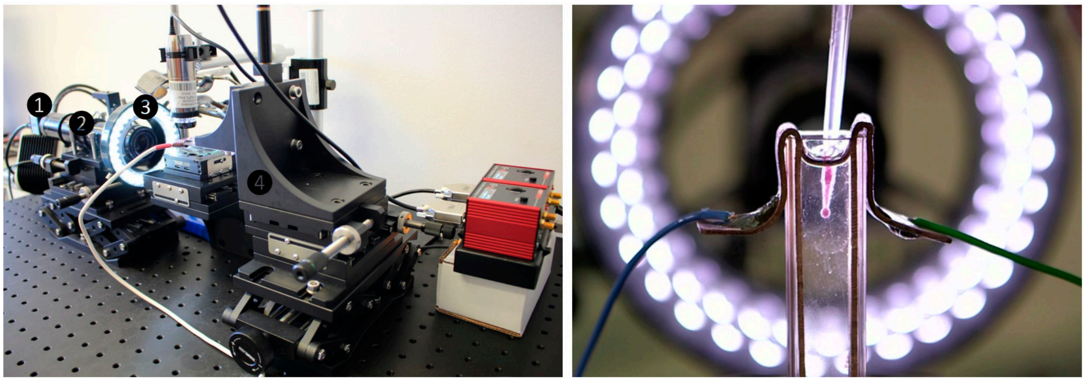

(**a**) (**b**)

Figure S5. Experimental set-up for the experiments on electric field-driven particle assembly of PS particles consisted of a CMOS camera ❶ mounted on a high-magnification zoom lens system ❷, a light source ❸, a sample cell placed on a mechanical x-y-z translational stage ❹, a voltage amplifier, an oscilloscope, and a PC for recording images (**a**). As sample cells, we used 10 mm × 10 mm plastic cuvettes (typically used for light spectroscopy) with two copper plates constituting electrodes (**b**).
